# Changes in the Effect of Heat on Mortality in the Last 20 Years in Nine European Cities. Results from the PHASE Project

**DOI:** 10.3390/ijerph121215006

**Published:** 2015-12-08

**Authors:** Francesca K. de’ Donato, Michela Leone, Matteo Scortichini, Manuela De Sario, Klea Katsouyanni, Timo Lanki, Xavier Basagaña, Ferran Ballester, Christofer Åström, Anna Paldy, Mathilde Pascal, Antonio Gasparrini, Bettina Menne, Paola Michelozzi

**Affiliations:** 1 Department of Epidemiology, Lazio Regional Health Service, Via di Santa Costanza 53, Rome 00198, Italy; m.leone@deplazio.it (M.L.); m.scortichini@deplazio.it (M.S.); m.desario@deplazio.it (M.D.S.); p.michelozzi@deplazio.it (P.M.); 2 Department of Hygiene and Epidemiology, Medical School, University of Athens, Athens 11527, Greece; kkatsouy@med.uoa.gr; 3 Department of Health Protection, National Institute for Health and Welfare (THL), Neulaniementie 4, PO Box 95, Kuopio 70701, Finland; timo.lanki@thl.fi; 4 Centre for Research in Environmental Epidemiology (CREAL), Doctor Aiguader 88, Barcelona 08003, Spain; xbasagana@creal.cat; 5 Department of Experimental and Health Sciences, Universitat Pompeu Fabra (UPF), Doctor Aiguader 88, Barcelona 08003, Spain; 6 Spanish Consortium for Research on Epidemiology and Public Health (CIBERESP), Melchor Fernández Almagro, 3–5, Madrid 28029, Spain; ballester_fer@gva.es; 7 FISABIO, Epidemiology and Environmental Health Joint Research Unit, Universitat Jaume I, Universitat de València, Valencia, 46020, Spain; 8 Occupational and Environmental Medicine, Department of Public Health and Clinical Medicine Umeå University, Umeå 90187, Sweden; christofer.astrom@umu.se; 9 Jozsef Fodor National Center of Public Health, National Institute of Environmental Health, Department of Biological Monitoring, Gyali ut 2–6, Po Box 64, Budapest 1097, Hungary; paldy.anna@oki.antsz.hu; 10 Department of Environmental Health (DSE), Institute de Veille Sanitaire, 12, rue du Val d’Osne, Saint Maurice 94415, France; m.pascal@invs.sante.fr; 11 Department of Social and Environmental Health Research, London School of Hygiene and Tropical Medicine, 15–17 Tavistock Place, London WC1H 9SH, UK; Antonio.Gasparrini@lshtm.ac.uk; 12 Department of Medical Statistics, London School of Hygiene and Tropical Medicine, Keppel Street, London WC1E 7HT, UK; 13 WHO Regional Office for Europe, European Centre for Environment and Health, Platz der Vereinten Nationen 1, Bonn D-53113, Germany; menneb@ecehbonn.euro.who.int

**Keywords:** heat, mortality, adaptation, attributable deaths, climate change, heat prevention plans

## Abstract

The European project PHASE aims to evaluate patterns of change in the temperature–mortality relationship and in the number of deaths attributable to heat in nine European cities in two periods, before and after summer 2003 (1996–2002 and 2004–2010). We performed age-specific Poisson regression models separately in the two periods, controlling for seasonality, air pollution and time trends. Distributed lag non-linear models were used to estimate the Relative Risks of daily mortality for increases in mean temperature from the 75th to 99th percentile of the summer distribution for each city. In the recent period, a reduction in the mortality risk associated to heat was observed only in Athens, Rome and Paris, especially among the elderly. Furthermore, in terms of heat-attributable mortality, 985, 787 and 623 fewer deaths were estimated, respectively, in the three cities. In Helsinki and Stockholm, there is a suggestion of increased heat effect. Noteworthy is that an effect of heat was still present in the recent years in all cities, ranging from +11% to +35%. In Europe, considering the warming observed in recent decades and population ageing, effective intervention measures should be promoted across countries, especially targeting vulnerable subgroups of the population with lower adaptive resources.

## 1. Introduction

Extreme temperatures are among the ten worst reported natural disasters of the past 40 years in terms of human lives lost globally [1]. In Europe, the death toll associated with such events is higher than for any other natural disaster, like floods and storms [1], and the adverse effect of high temperatures and heat waves across Europe has been extensively documented over the past decades in several multi-city time series studies [2,3]. Vulnerability to climate extremes is determined by the temperature–mortality relationship, which is heterogeneous across cities due to different climatic conditions, individual and population characteristics, and adaptation measures in place [4]. Local population characteristics include both individual (age, gender, income, education, ethnicity and social isolation) and contextual (population density, urban characteristics, socioeconomic, and access to health services) factors and may differ not only among different areas, but also over time within the same geographical area [5,6,7,8].

Climate change predictions indicate warmer temperatures and higher frequency of extreme events over Europe in future decades that are likely to increase heat-related impacts [9]. Southern European countries are experiencing more heat waves and higher temperatures, but northern areas are also vulnerable as they are warming at a faster rate [4]. 

Adaptation measures are crucial for reducing the current and future adverse impacts of climate change. The summer of 2003 was characterized by a very intense heat wave across most of Europe which had a considerable impact on health; with the increase in daily deaths during heat wave days compared to non-heat wave days between +20 and +110% [3]. In Europe, summer 2003 has changed individuals’ perception on heat-related health risks and has helped increase public awareness on climate-related threats. Furthermore, after 2003 the Ministries of Health of several European countries introduced public health plans with the aim of improving adaptation and reducing the adverse effects of hot weather [10,11]. These measures represent one of the few available climate adaptation strategies in the health sector but their effectiveness has not been formally evaluated. Temporal variations in the temperature–mortality relationship and in effect estimates have been used as an indirect evaluation of the potential benefits associated with the introduction of heat prevention measures. Evidence from the United States shows that, although mortality risk associated to heat exposure has been declining since 2000 compared with previous decades, it remains significant in most cities [12,13,14,15,16,17]. A similar declining trend has also been observed in European countries such as France [18], the Czech Republic [19], Italy [20,21] and Germany [22]. 

To date, an assessment of the potential changes in the impact of heat on mortality after the 2003 heat wave, considering the increase in temperatures recorded in the last decade and adaptation due to the introduction of heat plans, has not been carried out at the European level. Within the EU co-funded PHASE project, we estimated the temperature–mortality relationship and defined the number of heat-attributable deaths in nine European cities in two periods, 1996–2002 and 2004–2010. The aim of the paper is to evaluate whether heat continues to represent an important risk factor for mortality in European cities and describe the patterns of change in the elderly and in the adult population after the 2003 heat wave, which somewhat changed public awareness and public health attention on the issue.

## 2. Methods

### 2.1. Study Design and Population

The study was performed in nine European cities: Athens, Barcelona, Budapest, Helsinki, London, Paris, Rome, Stockholm and Valencia. Daily meteorological and mortality data were collected for the years 1996–2010. The study period was restricted to 6 months from April to September, as high temperatures during the warmer months were the exposure of interest. To investigate a possible change in the temperature–mortality relationship, two 7-year periods were compared. Specifically, period 1 (P1) comprised the years 1996–2002 and period 2 (P2) years 2004–2010. Summer 2003 was chosen as the cut off point, as it may have marked a change in the perception of health effects related to heat and a change in individual response mechanisms. Furthermore, in response to the 2003 heat wave, many European countries introduced heat prevention plans the following year. It was decided to exclude 2003 from the analyses because it was an unprecedented event with record high temperatures that had devastating effects on mortality and non-fatal health outcomes across most of Europe. Considering the before and after analysis, including 2003 in either two of the two 7-year periods would provide a distortion in the heat-related mortality estimates. 

### 2.2. Mortality Data

Mortality data are represented by daily counts for all causes, excluding external causes (natural mortality, ICD-9: 1–799), cardiovascular diseases (ICD-9: 390–459) and respiratory diseases (ICD-9: 460–519). Mortality counts were classified in 4 age groups (15–64 years, 65–74 years, 75–84 years, 85 plus years). It was decided to exclude children (0–14 age group) as this will be the focus of a separate study on the health effects of heat in the age group considering morbidity outcomes.

### 2.3. Environmental Data

All cities provided 3-hour meteorological data (air temperature and dew point temperature, wind speed, and barometric pressure at sea level) from the nearest airport weather station or from urban weather monitoring stations. Partners from each city a priori selected weather data to provide for the analyses based on data availability for the entire study period, quality of data and representativeness of average urban exposure (weather station included for each city are annotated in Table 1). A preliminary analysis was carried out to identify the best temperature exposure metrics among mean, minimum and maximum air temperature, and mean, minimum and maximum apparent temperature. The best fit was evaluated on the basis of the minimum cross-validated residuals and the Relative Risks (RR) from the different models considered as done by Barnett and colleagues [23]. Considering results for all cities, daily mean temperature (Tmean) was chosen as the best exposure variable (data not shown).

Air pollution data for each city were also provided by PHASE partners and were retrieved from urban monitoring networks; daily values were calculated using a standard methodology reported in previous European studies [24]. The maximum daily value of NO_2_ (lag 0–1) was chosen as an indicator of urban traffic pollution in all cities except for Barcelona where the 24-hour mean of PM_10_ was considered as NO_2_ was not available. This was included in the model as confounder variable.

### 2.4. Statistical Analysis

Time-series Poisson regression models were run separately in each city and in each period, in order to derive period- and city-specific temperature–mortality associations. The associations were modeled using a distributed lag non-linear model (DLNM), a flexible way to capture the complex non-linear and lagged dependencies of exposure–response relationships through two functions modeling exposure–response and lag-response relationships, respectively, and then combined in a cross-basis function [25]. Specifically, a quadratic B-spline for the exposure-response function with three internal knots and a natural cubic B-spline for the lag-response function with an intercept and three internal knots placed at equally spaced values in the log scale were selected. City-specific lag windows were considered to capture the different delay in the effect of heat in different populations. Modeling choices were tested in a sensitivity analysis changing both the type of spline, position and number of knots and extending the lag windows up to 40 days to consider longer delays in the effect of heat. The base model included a natural cubic B-spline of day of the year with equally spaced knots with 6 degrees of freedom (*df*) to control for seasonality, a natural cubic B-spline of time with 8 *df* to control long-term trends, and an indicator of day of the week and holidays. To account for the added effect of humidity with respect to mean temperature, relative humidity (%) was also introduced in the model as a quadratic B-spline with the same number of knots and lag as the exposure variable. The robustness of the choice of degrees of freedom (*df*) for the exposure variable, for long-term trend and for seasonality was also checked by comparing RRs derived from different combinations of *df* of each spline variable. Sensitivity analyses confirmed results from the main model.

Barometric pressure (lag 0–3) and wind speed (lag 0) were included as potential confounders on the basis of previous studies conducted in the same cities [2]. Both barometric pressure and wind speed were considered as linear terms. The maximum daily value of NO_2_ (lag 0–1) was also included as a confounder in all cities. 

The effect of high temperatures on mortality was expressed as the Relative Risks (RR) of daily mortality for increases in mean temperature from the 75th to 99th percentile of the summer period-specific distribution to capture the effects across a range of temperatures during the warm season. The analyses were run by cause of death and age groups. To assess the change in the effect of heat on mortality, the RRs were calculated separately for period 1 (P1) (1996–2002) and period 2 (P2) (2004–2010). To test for the statistical significance of the change in the effect the relative effect modification (REM) index was calculated as the ratio between the period-specific relative risks as defined in Stafoggia *et al.* [26]. The presence of effect modification was evaluated with a significance level of 0.05. 

Secondly, to estimate the impact of high temperatures on mortality city-specific attributable fractions (%) of death (AF) and number of deaths (AD) were calculated for the two periods. These attributable measures were estimated using the methodology developed by Gasparrini and Leone (2014) within the DLNM R framework, which takes into account the additional temporal dimension of the temperature–mortality association when providing risk estimates [27]. For each given day, the attributable fraction (AF) of mortality due to temperature was estimated by combining the risks on the given and previous days, according to the pre-defined lag window. The daily attributable number of deaths (AD) was calculated by multiplying the daily AF by the daily number of deaths. The total number of attributable deaths was given by the sum of the AD for all the days with temperatures between the 75th and 99th percentile of mean temperature. The total heat-attributable fraction represents the ratio between the total AD and total number of deaths. In order to estimate empirical confidence intervals, Monte Carlo simulations were implemented in the model. 

To estimate the potential change in the number of heat-related deaths between the two periods, we calculated the number of deaths attributable to heat considering the AF in each period by the number of deaths observed in period 2. This was done under the assumption that the underlying population exposed to heat does not change between periods but their response to heat has changed over time.

**Table 1:** Demographic characteristics, mean temperature distribution in the two periods between April and September and heat prevention plans in nine European cities.

	Population					Average Daily Death by Cause			Mean Temperature (°C) ^§^			Heat Prevention Plan
Cities	Period	Total	Percent Aged 65+	Percent Aged 75+	Period Specific Summer Death Rate *	Total	Respiratory	Cardiovascular	Average	75th Pctile	95th Pctile	Year of Activation, Coverage
Valencia	1996–2002	745,501	17.1	7.4	371.6	15.1	1.6	5.3	22.2	25.6	28.9	2004, national
	2004–2010	802,273	17.4	8.5	344.1	15.1	1.7	4.8	22.0	25.9	29.7	
Barcelona	1997–2002	1,507,563	21.9	10.0	441.0	36.3	3.3	12.5	20.4	23.6	26.8	2004, national, regional
	2004–2009	1,601,630	20.7	10.8	394.6	34.5	3.5	10.7	21.8	25.5	28.8	
Athens	1996–2002	3,288,193	15.7	6.3	409.5	73.6	5.5	36.1	24.3	28.4	34.0	n.a.
	2004–2010	3,283,460	16.7	7.8	426.5	76.5	7.8	34.8	24.3	28.3	33.2	
Rome	1996–2002	2,596,061	17.9	7.3	370.2	52.5	2.6	21.1	20.5	24.0	28.2	2004, national, regional
	2004–2010	2,679,363	20.5	9.1	366.6	53.7	3.1	21.3	21.1	25.0	29.4	
Budapest	1996–2002	1,817,370	17.0	7.2	679.4	65.9	1.9	32.7	18.1	21.7	28.2	2006, national
	2004–2010	1,706,734	18.3	8.7	638.2	59.5	2.5	28.9	18.5	22.1	28.3	
Paris	1997–2002	6,199,901	13.1	6.1	371.7	106.8	6.7	29.9	17.0	19.9	26.7	2004, national
	2004–2009	6,518,897	12.9	6.6	272.3	97.0	5.5	24.1	17.4	20.2	26.5	
London	1996–2002	7,163,486	12.7	6.0	376.4	147.3	23.3	57.9	15.2	17.9	24.1	2004, national
	2004–2010	7,613,413	11.7	5.7	296.0	123.1	16.4	43.3	15.6	18.1	24.6	
Stockholm	1996–2002	1,800,947	14.4	7.4	298.7	29.4	2.2	13.4	13.3	17.1	23.3	n.a.
	2004–2010	1,955,036	14.4	6.9	261.9	28.0	1.9	11.4	13.6	17.2	23.1	
Helsinki	1996–2002	942,492	11.4	5.0	332.5	17.1	1.5	7.4	12.6	16.9	22.7	n.a.
	2004–2010	1,010,775	12.7	5.6	312.1	17.2	0.9	6.9	13.0	17.0	23.8	

* (rate: per 100,000 inhabitants); ^§^ Weather stations: Valencia airport, Barcelona El Prat airport, Athens Eleftherios Venizelos airport, Rome Ciampino airport, Budapest Ferihegy airport, Paris Montsouris urban weather station, London Heathrow airport, Helsinki Vantaa airport, and Stockholm Bromma airport.

## 3. Results

The cities included in the study differed by socio-demographic characteristics, climate and heat prevention programs. Population size ranged from less than one million inhabitants in Valencia and Helsinki to more than seven million in London. Population structure was slightly different between cities, with the elderly population (65+ age group) ranging from 12% in London and Helsinki to 21% in Barcelona and Rome. In the recent period, the proportion of old and very old has increased in Athens, Budapest, Helsinki, Rome and Valencia, while in Barcelona and Paris this is only true for the 75+ age group. The dataset comprised of 1,322,844 deaths that occurred between April and September in the years 1996–2010 (excluding 2003). The average number of daily deaths varies considerably between cities, ranging from 135 deaths in London to less than 20 in Helsinki and Valencia (17 and 15 deaths per day on average, respectively). Period-specific death rates are higher in Budapest and lower in Stockholm and Helsinki, while when comparing the two periods, a reduction in rates seems to have occurred in all cities except for Athens. Cardiovascular deaths represented almost half of the total mortality, while respiratory deaths accounted for a smaller proportion (around 10%) (Table 1). A decline in the number of deaths was observed in all cities except for Rome and Athens. 

Heat plans are present in most cities except for Athens, Stockholm and Helsinki. Table S1 (Supplementary Material) summarizes the results of a survey conducted within the PHASE project on the main characteristics of the heat prevention plans in each city (www.phaseclimatehealth.eu). Some similarities are present among the different plans, and the common elements are early warning systems, near-real time health surveillance, information campaigns and prevention measures targeted to at-risk groups. 

**Figure 1:**
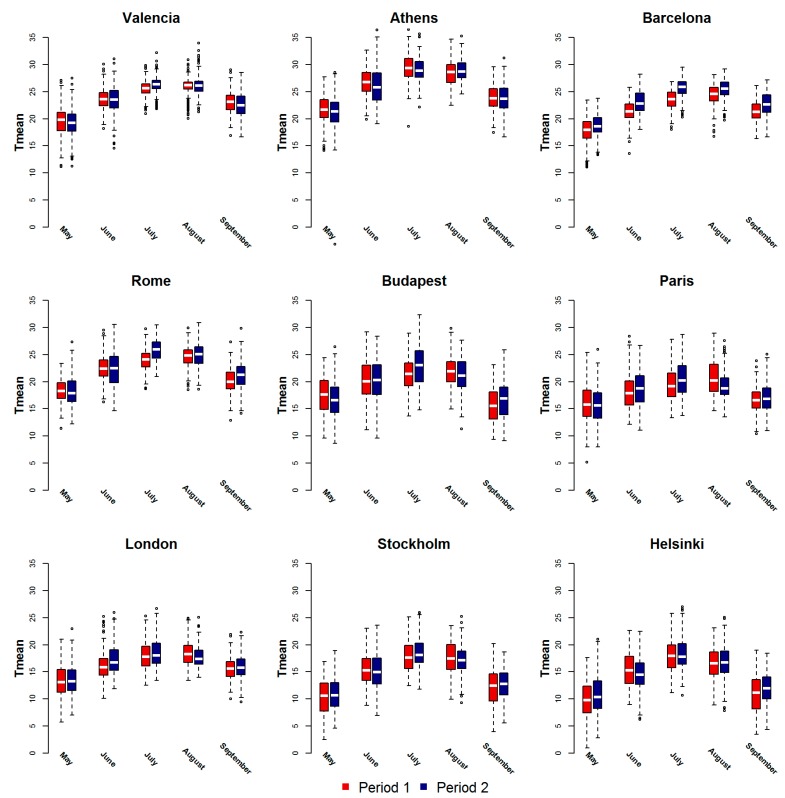
Boxplots of mean temperature (°C) distributions by month in the warm season, in the periods before (Period 1) and after 2003 (Period 2) in nine European cities 1996–2010.

Although the study period is too short to state whether we are actually observing a change in the trend in climatological terms, when comparing the two periods some differences are noteworthy in the context of our study. The local climatic characteristics differ somewhat between cities included in the study (Table 1, Figure 1). Cities in the Mediterranean are the warmest, while northern and continental European cities have lower average values. Figure 1 shows the mean temperature distribution by month in the two periods in study for each city. In the Mediterranean cities in the most recent period, temperatures have increased in July, August and September with higher extreme temperatures values. Differences were smaller in the other cities and occurring in the hottest month (July) and in September, suggesting a longer summer season. In several cities during the 20-year period considered, July seems to have become the warmest month. 

**Figure 2:**
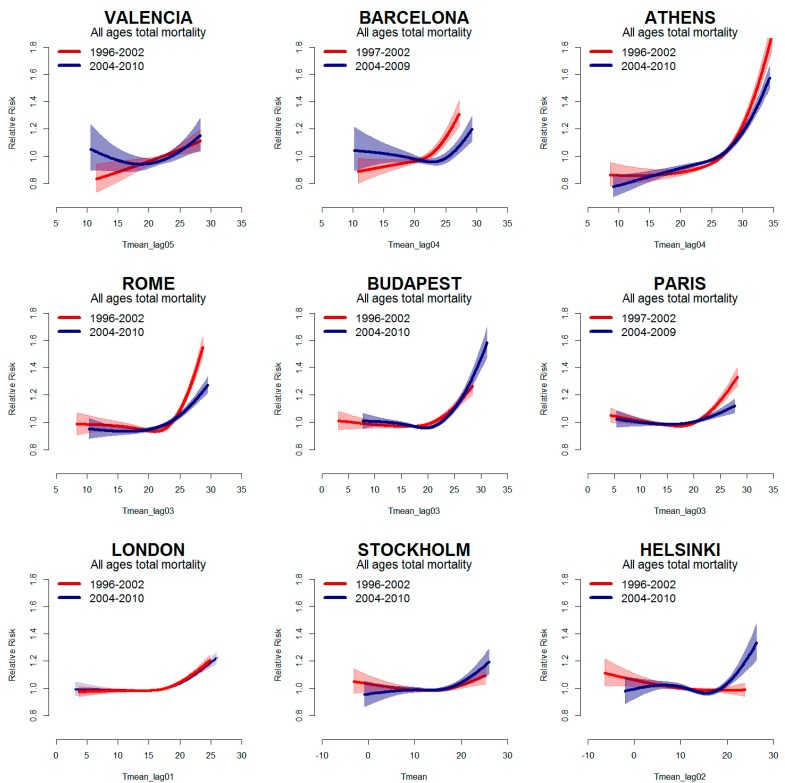
Mean temperature–mortality relationship (with 95% confidence intervals) in the years before 2003 (Period 1) (red line) and after 2003 (Period 2) (blue line) in Nine European cities. The x-axes show mean temperature (°C) (city-specific lag).

Figure 2 depicts the city-specific temperature–mortality relationship for all causes, estimated for the periods P1 (red line) and P2 (blue line). Curves show heat effects in all cities and in both periods, with an increase in mortality as temperatures increase. Only in Helsinki the curve showed no effect of high temperatures in P1. A reduction in the effect of high temperatures can be observed in the recent period in Paris, Rome and Athens (Figure 2, Table 2). In Barcelona, the curve shifts to the right in P2, but the slope remains essentially unchanged for high summer temperatures (Table 2). Conversely, an increase in the effect of high temperatures was observed in the northern and continental cities of Helsinki, Stockholm and Budapest, due to higher temperatures previously not observed. In London, although there seems to be a slight shift towards warmer summer temperatures (Table 1 and Figure 1), there is no evidence of a change in the risk of mortality related to high temperatures (Figure 2, Table 2). 

Results by cause (Table 2) show similar patterns of change for total, respiratory and cardiovascular causes of death in Athens, Paris and Rome, with a reduction in the effect in the second period. In Budapest, an increase in the effect of high temperatures was detected for all causes of death considered, although statistical significance was only reached for cardiovascular causes. In London, although a slight reduction in the effect of heat was observed for total mortality, there seems to be an increase in heat-related respiratory deaths. Although not statistically significant, it is interesting to note that in Stockholm and Helsinki the increase in the effect of high temperatures was mostly attributable to a rise in cardiovascular deaths.

**Figure 3:**
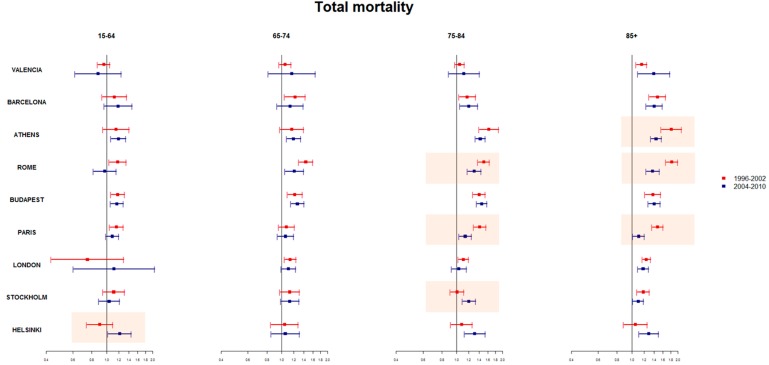
Estimated RRs (95% CI) for high temperatures and all cause daily mortality by age groups between the 75th and 99th percentile of mean temperature in the periods before 2003 (Period 1) (red line) and after 2003 (Period 2) (blue line). Nine European cities, 1996–2010. * orange boxes: significant REM index P1 and P2 effect estimate; Athens analyses by age group P1 = 1999–2002.

When considering age groups, the temporal changes observed in the all ages population are mostly attributable the change in heat-related deaths among the oldest age groups (75–84 and 85+ years) (Figure 3). In Paris and Rome, a significant reduction was observed for both 75–84 and 85+ years, while in Athens the reduction was only among the very old (85+ age group). In Helsinki, the increase in heat-related risk was observed in the 15–64 age group, the old and very old (75–84 and 85+), while in Stockholm, a significant increase was observed only in the 75–84 age group. 

A significant effect of heat in mortality was observed also in the younger age group (15–64 years) in several cities, which remains significant also in the recent period in Athens, Budapest and Helsinki. 

**Table 2:** Estimated Relative Risks (95% CI) for high temperatures and daily mortality for total, respiratory and cardiovascular causes in all age groups between the 75th and 99th percentile of mean temperature in the periods before 2003 (Period 1) and after 2003 (Period 2). Nine European cities, 1996–2010.

Cities	Period	All Causes			Respiratory Causes			Cardiovascular Causes		
		RR	95% Cl	P Value ^a^	RR	95% Cl	P Value ^a^	RR	95% Cl	P Value ^a^
Valencia	1996–2002	1.11	1.04–1.17		0.81	0.53–1.33		1.12	0.85–1.46	
	2004–2010	1.18	1.03–1.36	0.216	1.28	0.86–1.92	0.159	1.27	0.98–1.64	0.507
Barcelona	1997–2002	1.27	1.18–1.36		1.55	1.23–1.96		1.42	1.25–1.60	
	2004–2009	1.26	1.17–1.36	0.946	1.65	1.30–2.10	0.700	1.27	1.10–1.47	0.263
Athens	1996–2002	1.63	1.53–1.75		2.10	1.72–2.56		1.79	1.64–1.96	
	2004–2010	1.35	1.29–1.42	<0.001	1.42	1.23–1.63	0.001	1.53	1.43–1.64	0.006
Rome	1996–2002	1.53	1.45–1.61		2.04	1.65–2.53		1.72	1.59–1.86	
	2004–2010	1.27	1.19–1.35	<0.001	1.64	1.29–2.08	0.180	1.32	1.19–1.46	<0.001
Budapest	1996–2002	1.29	1.22–1.37		1.07	0.76–1.50		0.97	0.88–1.06	
	2004–2010	1.33	1.27–1.40	0.451	1.52	1.24–1.87	0.082	1.44	1.35–1.53	<0.001
Paris	1997–2002	1.31	1.24–1.37		1.72	1.43–2.06		1.25	1.15–1.37	
	2004–2009	1.11	1.06–1.17	<0.001	1.26	1.02–1.55	0.026	1.04	0.95–1.15	0.006
London	1996–2002	1.20	1.16–1.25		1.26	1.15–1.39		1.23	1.15–1.30	
	2004–2010	1.18	1.12–1.23	0.429	1.35	1.19–1.53	0.413	1.22	1.13–1.32	0.958
Stockholm	1996–2002	1.10	1.04–1.17		1.25	1.01–1.54		1.07	0.98–1.17	
	2004–2010	1.12	1.06–1.19	0.628	1.25	1.02–1.53	0.999	1.17	1.07–1.27	0.157
Helsinki	1996–2002	1.02	0.93–1.12		1.42	1.05–1.92		1.00	0.87–1.15	
	2004–2010	1.24	1.14–1.35	0.003	1.06	0.68–1.65	0.287	1.18	1.02–1.35	0.111

^a^ Significance test of effect modification based on REM index.

Table 3 shows the attributable risk fraction and the number of deaths attributable to mean temperatures comprised between the 75th and 99th percentile of summer distributions in each period. In terms of impact, a significant reduction in the number of deaths was observed in period P2 in Athens, Paris and Rome (985, 787 and 623 deaths, respectively). Results for the other cities showed no significant changes in terms of attributable risk (AR).

**Table 3:** Heat attributable risk fraction and deaths in the period P1 and P2 and change in heat attributable deaths in nine European cities, 1996–2010.

	P1 (Before 2003)			P2 (After 2003)			Change in Attributable Deaths	
Cities	AR%	95% Cl	Attributable Death	AR%	95% Cl	Attributable Death	Number of Deaths *	P Value
Valencia	**0.6**	(0.2–1.0)	112	**1.0**	(0.5–1.5)	194	82	0.343
Barcelona	**2.2**	(1.7–2.7)	896	**2.3**	(2.0–2.6)	870	19	0.916
Athens	**3.4**	(3.0–3.8)	3200	**2.4**	(2.1–2.7)	2343	−**985**	**0.005**
Rome	**2.8**	(2.6–3.1)	1900	**1.9**	(1.6–2.3)	1321	−**623**	**0.006**
Budapest	**1.5**	(1.3–1.8)	1291	**1.7**	(1.3–2.0)	1302	136	0.597
Paris	**1.6**	(1.3–1.8)	1846	**0.8**	(0.6–1.1)	890	−**787**	**0.005**
London	**0.8**	(0.6–0.9)	1486	**0.7**	(0.5–0.9)	1080	−162	0.554
Stockholm	**0.7**	(0.3–1.0)	247	**0.7**	(0.4–1.0)	252	17	0.889
Helsinki	**0.1**	(−0.5–0.6)	14	**0.9**	(0.4–1.4)	202	188	0.115

* calculated assuming same population is exposed but with different AR.

## 4. Discussion

Findings show heat still has a considerable significant effect on mortality in the European cities included in the study. Moreover, when comparing the effect estimates of heat on mortality between the two periods, the study shows different patterns of change among the cities included. A significant reduction in the attributable number of deaths was observed in Athens, Rome, Paris and, marginally, in London. In the other cities, no change or an increase in the number of deaths attributable to heat was observed. In particular, an increase in the effect of high temperatures on mortality was observed in cooler cities where extreme temperatures in recent years have risen, exposing local populations to unusual summer heat. 

Different factors need to be mentioned when explaining the temporal changes in the association between heat and mortality. Firstly, changes in the exposure clearly play an important role. The warming observed in most European cities in the last decades, as summarized in the latest IPCC report [4], may have altered the temperature–mortality relationship, especially in areas where the local populations were not prepared to cope with higher temperatures. This may be the case for the northern European cities of London, Helsinki and Stockholm. 

In Barcelona, temperatures have increased in recent years (+2 °C in 75th and 99th percentiles in the period 2006–2010), and from the inspection of the temperature–mortality curve, we observe a shift to the right of the curve in the second period, suggesting adaptation to milder summer temperatures. However, when considering the extreme temperatures there seems to be no change in heat-attributable deaths, suggesting the impact of extreme temperatures remains unchanged or acclimatization to these extreme exposures has not occurred. A specific study on the turning point of the curves would have helped identify the temperature above which mortality increases and how these have changed over time.

Another important factor that might have influenced the temporal pattern of change in the effect of heat on mortality are the heat-response measures undertaken at the national and local level. Heat-adaptation measures, together with improvements in infrastructures and health care services can have beneficial effects reducing the impact of heat on mortality [12,13,14,15,16,17,18,19,20,21,22]. We observed a significant decrease in the impact of high temperatures on mortality in Rome and Paris. Results from the survey carried out within PHASE, show that in both cities, heat plans were implemented after 2003 and include the core elements identified by WHO [28], such as warning systems, prevention measures, health surveillance and definition of susceptible subgroup. However, they differ somewhat in terms of spatial coverage, management and institutions involved, reflecting local and national policy in terms of emergency response, public health and climate change strategies (Table S1). Although evidence on the effectiveness of specific intervention measures is lacking [29,30], it is plausible that heat prevention programs targeted to the elderly and specific susceptible groups have enhanced population awareness and improved response to heat waves in recent years in Italy and France [18,20].

Evaluation of public health programs is challenging, particularly in the specific topic of heat prevention due to the heterogeneity of local weather patterns over time, the complexity of the heat-related prevention programs and the confounding factors that vary over time. Due to the impossibility of conducting randomized trials in this context, before and after studies represent a feasible approach to evaluate variations in the impact of heat on mortality after implementation of heat response plans [29,30]. These studies have also been used in other public health contexts [31]. Furthermore, it is worth mentioning that research on the effectiveness of measures put in place is very sparse, but it is of great importance to identify which measures are most appropriate and effectively target the limited public health resources.

In both periods, Athens was the warmest city and one of the cities with the highest effect estimates. The reduction in the impact of heat in the recent period was observed in the oldest population subgroup also in Athens. This change has no clear attribution in terms of public health adaptation measures or population adaptive capacity since the city has no prevention plan in place. A speculative reason could be that after 2003 individual awareness and adaptive capacity could have changed in response to the great media attention in most European countries. 

The use of air conditioning, as suggested by several US studies, can also influence the response to heat reducing the health effects of temperature [12,15,17,32,33]. However, a recent study suggests that in several areas of the US, air conditioning has reached market saturation in the last decade and thus may play a minor role in reducing the effect of heat [15]. To date, a similar study on European cities has not been carried out. Moreover, air conditioning in housing as well as in public structures is less common and heterogeneous among European countries and further research on this topic is needed to address the issue.

Previous studies identified several individual characteristics that modify the association between heat and mortality, in particular demographic and socio-economic factors [26,32,33,34,35,36,37,38]. Furthermore, it is important to consider that the pool of susceptible subgroups change over time and this aspect needs to be considered when describing temporal variations in the impact of heat on health outcomes. Population is ageing in many European cities, thus potentially inflating the pool of subjects vulnerable to heat, as it is well known that mortality risk due to temperature increases with age and gender [5,8]. Although evidence is less clear for gender differentials [5,8,26,34,36], the higher number of elderly women and the different susceptibility factors by gender may be associated with a higher risk among elderly females. However, in several cities where a reduction in the effect was observed in recent years, the decrease in mortality occurred mainly in the over 75 year old population for both cardiovascular and respiratory causes suggesting the potential role of prevention plans that are focused on these subgroups [18,20]. Similarly, in US cities, Bobb *et al.* [15] found a more rapid decline in heat-related mortality in the elderly between 1987 and 2005. This was attributed to heat prevention programs targeting mostly this age group, or to other factors, such as a greater awareness of risks of extreme heat or a shift from heat-related mortality to heat-related morbidity. It is interesting to note that, among the 65–74 years olds, the reduction was minimal, or not observed, suggesting more has to be done for this group in current heat prevention programs. 

Considering pre-existing chronic disease, the effect of heat on cardiovascular and respiratory causes found in our study confirms the higher vulnerability to heat in patients with these conditions (e.g., myocardial infarction survivors and COPD) as reported in previous [5,26,36,37]. This has important consequences in future years as the prevalence of these diseases are growing not only among the elderly but also in the younger population [39,40]. The rising risk of heat-related mortality in some cities can be ascribed to different pre-existing disease not considered in this study such as diabetes, neurologic and psychiatric diseases that are also on the rise among the adult population in Europe. Findings can be translated into a recommendation for public health to intensify prevention measures on younger population during hot weather especially towards those vulnerable due to pre-existing chronic health conditions.

European countries have been facing socio-economic challenges in recent years such as the economic recession, rising unemployment rate, population growth in urban areas, and increasing poverty and social exclusion [41]. The economic recession may have worsened citizens’ social and economic conditions thus reducing their individual adaptive capacity. Economic restrictions have also had an impact on healthcare systems, with an abatement of expenditures for hospital equipment, personnel, prevention programs and other healthcare costs thus affecting the capacity of health systems to respond to emergencies [10,11,42]. 

## 5. Conclusions

Climate change is recognized as the biggest global health threat of the 21st-century. Our study shows that heat still has a considerable impact on mortality. Considering the increase in the frequency and intensity of heat waves predicted under the different climate change scenarios, it seems imperative that public health adopts a central role in promoting preparedness and response to climate change among European populations. The implementation of intervention measures, specifically targeted to vulnerable subgroups with limited adaptive capacity, will help enhance adaptation and reduce heat-related risks.

## Supplementary Material

**Table S1:** Main components of the Heat Plans in nine European cities included in the PHASE project.

City	Year of Activation	Level (Responsible Body)	Warning System (Model Type)	Susceptible Subgroups	Information Campaign, Professional Training Programmes	Emergency Actions During Heat Waves	Health Surveillance (Indicator; Timing)	Evaluation of Preventive Measures
Valencia	2004	national (Ministry of Health)	Yes (Maximum apparent temperature-mortality regression model)	elderly, chronically ill, pharmaceutical treatments, persons with cognitive diseases	general population, susceptible groups, athletes, social and health workers	reinforced communication to health professionals, social services, general population; additional measures for targeted groups (home visits by the social workers, telemonitoring)	Yes (mortality; daily)	
Barcelona	2004	national (Ministry of Health) regional (Dep.t of Health of Autonomous region)	Yes (Threshold model based on temperature (Tappmax)-mortality relationship)	children, elderly, chronically ill, people in pharmaceutical treatment, disabled, isolated people, pregnant women	general population, susceptible groups, athletes, social and health workers	opening cooling spaces, postponing non-urgent surgery, protected discharges	Yes (mortality; daily)	
Athens			No		general population, susceptible groups		No	
Rome	2004	national (Ministry of Health) regional (regional Department of Health Care)	Yes (Threshold model based on temperature (Tappmax)-mortality relationship; Air mass based model)	elderly, isolated people, chronically ill, people in pharmaceutical treatments, pregnant women, workers in construction, transport and mining sectors	general population, susceptible groups, social and health workers	opening cooling spaces, postponing non-urgent surgery, protected discharges, mobilise community and voluntary support; additional measures for targeted groups (home visits by the family doctors)	Yes (mortality, daily; emergency visits, weekly for emergency visits)	before and after study comparing the health effects of high temperatures (Schifano 2012) evaluation of GPs surveillance comparing tempertuare effects on sruveilled/not surveilled (Bargagli 2011)
Budapest	2006	national (National Health Service)	Yes (climatological tempertuare threshold model)	people in health facilities, homeless people	general population	reinforced social care services, intensified air conditioning in health care facilities, providing water in public areas especially to homeless people	Yes (mortality, ambulance calls; daily)	
Paris	2004	national (Ministry of Health)	Yes (Threshold model based on temperature (tmin and Tmax)-mortality relationship)	children, elderly, chronically ill, people with cognitive troubles, imprisoned people, phamaceutical treatments, disabled, isolated people, drug/alcohol abusers, homeless people	general population, susceptible groups, social and health workers	activation of free help line, opening cooling spaces, emergency plans in hospitals and retirement homes, reinforce the patrols of the Samu Social (for homeless people)	Yes (mortality, emergency visits; daily)	before and after study comparing the health effects of heatwaves (Fouillet 2008)
London	2004	national (National Health Service)	Yes (climatological tempertaure threshold models -Tmax Tmin)	children, elderly, chronically ill, disabled, drug/alcohol abusers	general population, susceptible groups, Muslims during Ramadan, social and health workers	media alerts, support organisations to reduce unnecessary travel, review safety of public events, mobilise community and voluntary support	Yes (mortality, emergency visits and NHS 111 calls; weekly, during heat waves daily)	
Stockholm					general population			
Helsinki					general population, health workers			
